# 534 If You Build It, They Will Come: Everyday Burn Clinic Enhanced Access to Care

**DOI:** 10.1093/jbcr/irad045.131

**Published:** 2023-05-15

**Authors:** Casey O'Rourke, Megan Gernt, Kristen Burton-Williams

**Affiliations:** Rhode Island Burn Center, Providence, Rhode Island; Rhode Island Hospital,Providence, Rhode Island; Rhode Island Burn Center, Providence, Rhode Island; Rhode Island Hospital,Providence, Rhode Island; Rhode Island Burn Center, Providence, Rhode Island; Rhode Island Hospital,Providence, Rhode Island

## Abstract

**Introduction:**

Prior to 2020, our small burn center had 1x/wk adult burn clinic and 1x/wk pediatric burn clinic. This schedule did not allow for optimal access to care for the geographic area our burn center serves. Also, our inpatient daily census was steadily increasing, and we identified many barriers to discharge for our acute burn survivors that would help to justify to hospital administration the need for increasing the frequency of our burn clinic to daily. These barriers to discharge included decreased availability of visiting nurse agencies for dressing changes, poor quality of rehabilitation services through visiting nurse agencies, and comfort and/or competence with family and/or caregivers being able to provide quality needed burn care.

**Methods:**

Our burn leadership team developed a business plan for an everyday burn clinic to provide to hospital administration. This daily burn clinic would allow for both adult and pediatric burn survivors to be seen Monday-Friday, provide rehabilitative services a space within our burn clinic, and availability of daily dressing changes in burn clinic. The business plan was reviewed by hospital administration, meetings were held with hospital administration, rehabilitation department leadership and ancillary services leadership to help validate the need for the daily burn clinic.

**Results:**

Hospital administration approved our daily burn clinic in July of 2020 allowing for improved outpatient dressing and wound management, easy access to quality burn rehabilitation, translating to decreased length of stay. Communication was sent to all area emergency rooms, urgent cares, pediatrician offices and primary care offices to inform them of the increased availability of the burn clinic. This communication included the American Burn Association transfer criteria, a referral form to facilitate access to our burn clinic, and a simple algorithm for assistance with triaging burn care. Prior to the everyday burn clinic in 2019, we were seeing 1,155 outpatient visits annually and in 2021 we saw 1,728 visits in our burn clinic. This is a 150% increase in outpatient burn clinic visits in two years.

**Conclusions:**

Increasing accessibility to outpatient care for the burn survivor can benefit many aspects in their journey to recovery as well as support the surrounding community in quick access to quality burn care. Burn survivors benefit from shorter hospital stays with the availability of a daily burn clinic for ongoing dressing management while maintaining quality rehabilitation services to maximize their functional outcomes.

**Applicability of Research to Practice:**

Data could be collected to evaluate the true impact the creation of the daily burn clinic had on burn survivors’ length of stay. Also, data can be collected regarding the number of burn survivors treated at other local emergency rooms and/or urgent care facilities prior to the creation of the daily burn clinic to evaluate the efficacy of the communication provided to the community.

